# Unraveling Non‐Suicidal Self‐Injury Functions Through Network Analysis and Revealing Their Triangular Relationship With Psychosocial Factors and Personality Traits

**DOI:** 10.1002/brb3.71716

**Published:** 2026-08-26

**Authors:** Huiru Yan, Hao Yan, Mingzhu Li, Yuqi Ge, Yaoyao Sun, Chuan Shi, Chao Wu, Weihua Yue, Xiao Zhang

**Affiliations:** ^1^ Peking University School of Nursing Peking University Beijing China; ^2^ Peking University Sixth Hospital, Peking University Institute of Mental Health NHC Key Laboratory of Mental Health (Peking University), National Clinical Research Center for Mental Disorders (Peking University Sixth Hospital) Beijing China

**Keywords:** non‐suicidal self‐injury, emotion regulation, network analysis, functional heterogeneity, personalized treatment

## Abstract

**Background:**

Non‐suicidal self‐injury (NSSI) imposes a significant burden on adolescents, making it essential to understand its underlying functions to develop effective interventions. This study aims to investigate the NSSI functional network and clarify the “psychosocial factors‐personality‐behavior” chain to inform the development of interventions for NSSI.

**Methods:**

A total of 265 psychiatric inpatients aged 16–25 years with NSSI participated in this cross‐sectional study. Network analysis was used to examine relationships among NSSI functions, and mediation analysis explored the pathways from psychosocial factors and personality to these functions.

**Results:**

Five stable NSSI functional communities were identified, including internal emotion regulation (IER), psychological pain avoidance (PPA), social influence (SI), suicide resistance (SR), and sensation seeking (SS). The core function of IER exhibited the highest centrality. Psychosocial factors, including health adjustment (*β_IER_ =* 0.450, *β_PPA_ =* 0.303, *β_SI_ =* 0.322, *β_SS_ =* 0.130), interpersonal stress (*β_IER_ =* 0.228, *β_PPA_ =* 0.196, *β_SI_ =* 0.301), neuroticism (*β_IER_ =* 0.205, *β_PPA_ =* 0.165), childhood emotional mistreatment (*β_IER_ =* 0.204, *β_PPA_ =* 0.195), openness to experience (*β_SI_ =* 0.146), conscientiousness (*β_SR_ =* 0.066), and extraversion (*β_SR_ =* −0.079) were significantly associated with various NSSI functions. Neuroticism mediated most of the pathways connecting psychosocial factors to NSSI functions such as IER, PPA, and SI.

**Conclusions:**

Internal emotion regulation was the central driver among five functions of NSSI. Neuroticism played a key mediating role in the link between psychosocial factors and NSSI functions. Tailored interventions targeting emotion regulation are essential.

## Graphical abstract

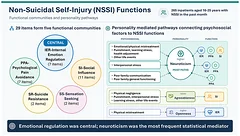

## 1 Introduction

Non‐suicidal self‐injury (NSSI) refers to the behaviors of an individual intentionally and repeatedly changing or harming their own body without a clear intention of suicide (Nock et al. 2006). There are many ways of NSSI, including cutting, carving, burning, swallowing dangerous goods, striking the head, and poking body parts with needles or sharp objects, which causes significant tissue damage (Ammerman et al. 2019). The incidence of NSSI among adolescents has been increasing in recent years (Mercado et al. 2017), having a terrible impact on social functions and increasing the risk of suicide (Mars et al. 2014; Ribeiro et al. 2016). While NSSI places a heavy burden on adolescents, understanding its underlying functions is critical for advancing knowledge of the mechanisms that maintain this behavior.

Individuals partake in NSSI for a multitude of reasons. However, the relationships between the conceptual frameworks and empirical evidence of its various functions are not well‐defined. Our review of extant research delineates its primary functions as emotion regulation, social/interpersonal influence, sensation seeking, anti‐suicide, etc. Emotion regulation function is defined as reducing the arousal of negative or disgusted emotion (Andover and Morris 2014). The interpersonal influence function believes that self‐harm can influence the interpersonal environment or control others, including seeking help, avoiding abandonment, or attempting to make others care more about oneself (Klonsky 2007). The sensation‐seeking function is defined as generating excitement through self‐injury and is regarded as an automatic positive reinforcement (Klonsky 2007; Selby et al. 2014). The anti‐suicide function considers NSSI as a current strategy to resist suicide ideation or attempts (Kraus et al. 2020). From this perspective, self‐injury can be seen as a means of expressing suicidal thoughts without risk of death and a substitute or compromise for suicide. The different functions of NSSI not only predict the persistence of future NSSI (Pollak et al. 2020) but also have different impacts on treatment, prognosis, and suicide risk (Klonsky and Glenn 2009; Klonsky and Olino 2008; Nock and Prinstein 2005; Paul et al. 2015). In this study, we aimed to explore the functional network of NSSI, identify the central function within this network, and ascertain the correlation patterns with other functions.

Understanding the underlying functions of NSSI is crucial, and it is equally important to further clarify the psychosocial factors associated with NSSI. The developmental psychopathological model (Yates 2004), based on developmental organizational theory, explains NSSI from a developmental perspective. Developmental organizational theory suggests that individuals gradually develop five adaptive capacities during development, including positive motivations and expectations for building relationships with others, self‐esteem and self‐worth, positive adaptation and specific skills, a strong emotional base, and the capacity to understand the rules of social reciprocity. According to the developmental psychopathological model, long‐term chronic stress, such as traumatic experiences within the family, may impair these adaptive capacities. As a result, individuals may experience self‐isolation and negative self‐representations, such as self‐blame and impulsiveness, and may use their bodies to regulate emotions or cope with interpersonal difficulties. In this context, NSSI is viewed as a compensatory coping strategy that serves emotional, interpersonal, and other functions (Yates 2004). Therefore, factors such as childhood experiences and family environment are highly likely to be associated with different functions of NSSI. Besides, previous research suggests that personality traits are shaped by environmental influences and subsequently affect clinical phenotypes (Jiang et al. 2024). There was also a close correlation between personality traits and NSSI (Kiekens et al. 2015). Thus, psychosocial factors and personality may have complicated interaction, which can affect NSSI functions. In present research, potential psychosocial factors related to NSSI include family functioning, childhood trauma, and adolescent life events (Kaess et al. 2020; Liu et al. 2018; Y. Wang et al. 2022). Possible personality‐related factors include the big five personality traits (Goddard et al. 2021; Yu et al. 2024). But most studies are confined to correlations between a few factors, with a paucity of research delineating the complex chain of “psychosocial factors‐personality‐behavior”. This gap has impeded the development of effective, targeted psychosocial and behavioral interventions. Consequently, we have assembled a substantial sample of NSSI cases across various diagnostic categories to investigate the factors influencing NSSI functions and to elucidate the interplay among psychosocial factors and NSSI functions. Additionally, by examining NSSI in East Asian populations, this study enhances the academic understanding of the psychosocial mechanisms of NSSI within this region.

In this study, we proposed three hypotheses: (a) emotion regulation plays an important role in the NSSI functional network; (b) psychosocial factors such as life events, childhood abuse, and family functioning are associated with NSSI functions; and (c) the influences of psychosocial factors on NSSI function are mediated by personality traits.

## 2 Materials and Methods

### 2.1 Participants

This study employed a cross‐sectional design and collected data via self‐reported questionnaires. Patients were recruited from a psychiatric hospital in Beijing, China. The existing mental disorder was diagnosed by two senior doctors within the first week of admission to the clinic. Of the 388 hospitalized patients initially screened, 305 met the inclusion criteria. To minimize recall bias in self‐reported self‐harm assessment, we further included only those who had engaged in self‐harm behavior within the past month. We excluded all patients with only suicidal ideation or behavior but with no NSSI. Our final sample was comprised of 265 inpatients. This study was approved by the ethics committee of the Peking University Sixth Hospital (2021‐39). All participants signed a written informed consent form.

### 2.2 Assessment of NSSI

The functions of NSSI were assessed by the Ottawa self‐injury inventory‐function subscale (OSI‐F) (Zhang et al. 2015), a self‐reported inventory including 29 items to investigate the reason (e.g., to relieve feelings of sadness or feeling “down”) for engaging in NSSI in the last month and corresponding degree ranging from 0, never a reason, to 4, always a reason. This scale could mirror the functions from past reviews and was valid and reliable in clinical samples of adolescents (Nixon et al. 2015).

### 2.3 Assessment of Psychosocial Features

The Adolescent Self‐Rating Life Events Checklist (ASLEC) (X. D. Wang 1999), covering punishment, loss, interpersonal stress, learning stress, health adjustment, and other events, was used to evaluate life events experienced in the past 12 months and their impact. The Family Assessment Device (FAD) (X. D. Wang 1999) was adopted to assess family functions, including problem‐solving, communication, roles, affective responsiveness, affective involvement, behavior control, and general functioning. The Childhood Trauma Questionnaire (CTQ) (Zhao et al. 2005) was used to investigate individuals’ experiences of childhood abuse, including emotional mistreatment, physical mistreatment, sexual abuse, emotional negligence, and physical negligence. These scales indicate a poorer state with higher scores. Personality traits were assessed by the NEO Five‐Factor Inventory (NEOFFI) (Pervin et al. 2003), including neuroticism, extraversion, openness to experience, agreeableness, and conscientiousness. More details about the assessment tools are described in Supplementary material.

### 2.4 Statistical Analysis

#### 2.4.1 Demographic and Descriptive Analysis

Demographic, NSSI‐related variables, and psychosocial features were assessed with descriptive means and percentages as warranted. The independent samples t‐tests and chi‐square tests were performed to compare differences between males and females.

#### 2.4.2 The Functional Network Analysis

The network analytical procedure was performed in the R language using the qgraph package (Epskamp et al. 2012). Select 29 NSSI functions of OSI‐F as nodes, and the edges between nodes represent correlations. To identify essential functions, we examined centrality through three indices, strength, closeness, and betweenness (Kenett et al. 2011). Strength represents the sum of the absolute values of the edges’ weights of other nodes connected to the node. Closeness refers to the reciprocal of the sum of the shortest paths from the node to other nodes. Betweenness represents the number of times the node appears on the shortest path between the other two nodes. The larger the values of the above indices, the stronger the node centrality and the more important its role in the network. The correlation stability coefficients (CS‐coefficient) of edge and strength generated by the case‐dropping bootstrap method were used to quantify the stability of the centrality of a network, which should not be lower than 0.25 (Epskamp et al. 2018).

#### 2.4.3 Correlation and Regression Analysis

The Pearson correlations were performed to determine the inter‐relationships between variables. Multivariate regression analysis included all variables significantly (*p* < 0.05) correlated with each NSSI function using the step‐wise regression method. We also set gender, age, and dummy variables of clinical diagnosis as covariates to control potential effects.

#### 2.4.4 Mediation Analysis

To clarify the intricate factors‐personality‐behavior chain of NSSI functions, we constructed mediating models with external psychosocial factors as independent variables, personality traits as mediators, and NSSI functions as dependent variables. Age, gender, and diagnosis were included as covariates to control for their potential influence on the outcomes. The indirect effects were estimated via a bias‐corrected bootstrapping method with 5000 resamples. If the 95% confidence interval (CI) did not overlap with zero, it indicated that the mediating effect was significant.

## 3 Results

### 3.1 Demographical Information

Among the sample of 265 participants, 79.2% were female (N = 210), and 20.8% were male (N = 55), with a mean age of 18.74 years (SD = 2.37). There were 55.8% of participants diagnosed with depressive disorder, 30.2% with bipolar disorder, and 14.0% with eating disorder (Table 1). Repeated NSSI was defined as the individual who has 5 or more days engaged in NSSI in the past year, which meets the recommended diagnostic criteria for NSSI in DSM‐5. Occasional NSSI was defined as the individual engaged in NSSI 1 to 4 times in the past. Among the entire sample, 64.9% reported repeated NSSI.

**TABLE 1:** Socio‐demographic features of participants.

Variables	Males (N = 55)	Females (N = 210)	*t / X^2^*	*p*‐value
**Age**	18.53 ± 2.37	18.79 ± 2.37	−0.732	0.465
**Years of education**	11.89 ± 1.87	12.27 ± 2.49	−1.245	0.216
**Functions total score**	36.95 ± 29.65	36.33 ± 23.10	0.143	0.886
**Diagnosis**			6.179	0.046
Depressive disorder	34(23.0%)	114(77.0%)		
Bipolar disorder	19(23.8%)	61(76.3%)		
Eating disorder	2(5.4%)	35(94.6%)		
**Frequency of NSSI**			0.290	0.590
Repeated	34(19.8%)	138(80.2%)		
Occasional	21(22.6%)	72(77.4%)		
**ASLEC^a^**				
Punishment	10.51 ± 7.97	10.03 ± 6.44	0.463	0.644
Loss	3.38 ± 3.68	3.39 ± 3.85	−0.007	0.995
Interpersonal stress	11.87 ± 6.62	12.44 ± 5.60	−0.581	0.563
Learning stress	10.16 ± 5.70	10.89 ± 5.54	−0.856	0.393
Health adjustment	6.71 ± 3.33	7.29 ± 4.36	−1.075	0.285
Others	7.60 ± 4.94	7.43 ± 3.96	0.232	0.818
**FAD^b^**				
Problem solving	2.42 ± 0.39	2.31 ± 0.50	1.584	0.114
Communication	2.65 ± 0.43	2.61 ± 0.53	0.634	0.526
Roles	2.43 ± 0.37	2.38 ± 0.43	0.697	0.486
Affective responsiveness	2.60 ± 0.52	2.62 ± 0.63	−0.156	0.877
Affective involvement	2.51 ± 0.37	2.57 ± 0.58	−0.754	0.452
Behavior control	2.45 ± 0.3	2.47 ± 0.35	−0.374	0.709
General functioning	2.43 ± 0.49	2.44 ± 0.57	−0.010	0.992
**CTQ^c^**				
Emotional mistreatment	11.18 ± 4.13	11.52 ± 5.05	−0.457	0.648
Physical mistreatment	8.87 ± 5.07	7.69 ± 4.09	1.604	0.113
Sexual abuse	5.98 ± 2.21	6.35 ± 3.38	−0.980	0.329
Emotional negligence	15.11 ± 4.21	14.21 ± 5.26	1.329	0.187
Physical negligence	8.76 ± 3.25	8.71 ± 3.34	0.108	0.914
**NEOFFI^d^**				
Neuroticism	36.65 ± 6.25	37.62 ± 6.19	−1.027	0.305
Extraversion	22.24 ± 5.74	21.84 ± 7.00	0.436	0.663
Openness to experience	33.47 ± 5.65	33.64 ± 6.64	−0.174	0.862
Agreeableness	31.18 ± 5.68	32.79 ± 5.09	−2.034	0.043
Conscientiousness	28.45 ± 6.23	29.14 ± 7.50	−0.626	0.532

*Note*: NSSI, non‐suicidal self‐injury; ASLEC, Adolescent Self‐Rating Life Events Check List; FAD, Family Assessment Device; CTQ, Childhood Trauma Questionnaire; NEOFFI, NEO Five Factor Inventory. a, the score ranges of ASLEC dimensions are: punishment (0∼35), loss (0∼15), interpersonal stress (0∼25), learning stress (0∼25), health adjustment (0∼20), others (0∼20), higher scores indicate a greater impact of negative life events. b, the score range of each FAD dimension is 1–4; higher scores indicate poorer family functioning. c, the score range of each CTQ dimension is 5–25; higher scores indicate more severe childhood trauma. d, the score range of each NEOFFI dimension is 12–60, higher scores indicate higher levels of the corresponding personality trait.

### 3.2 Network Analysis

#### 3.2.1 Network Estimates and Centrality Analysis

**FIGURE 1:**
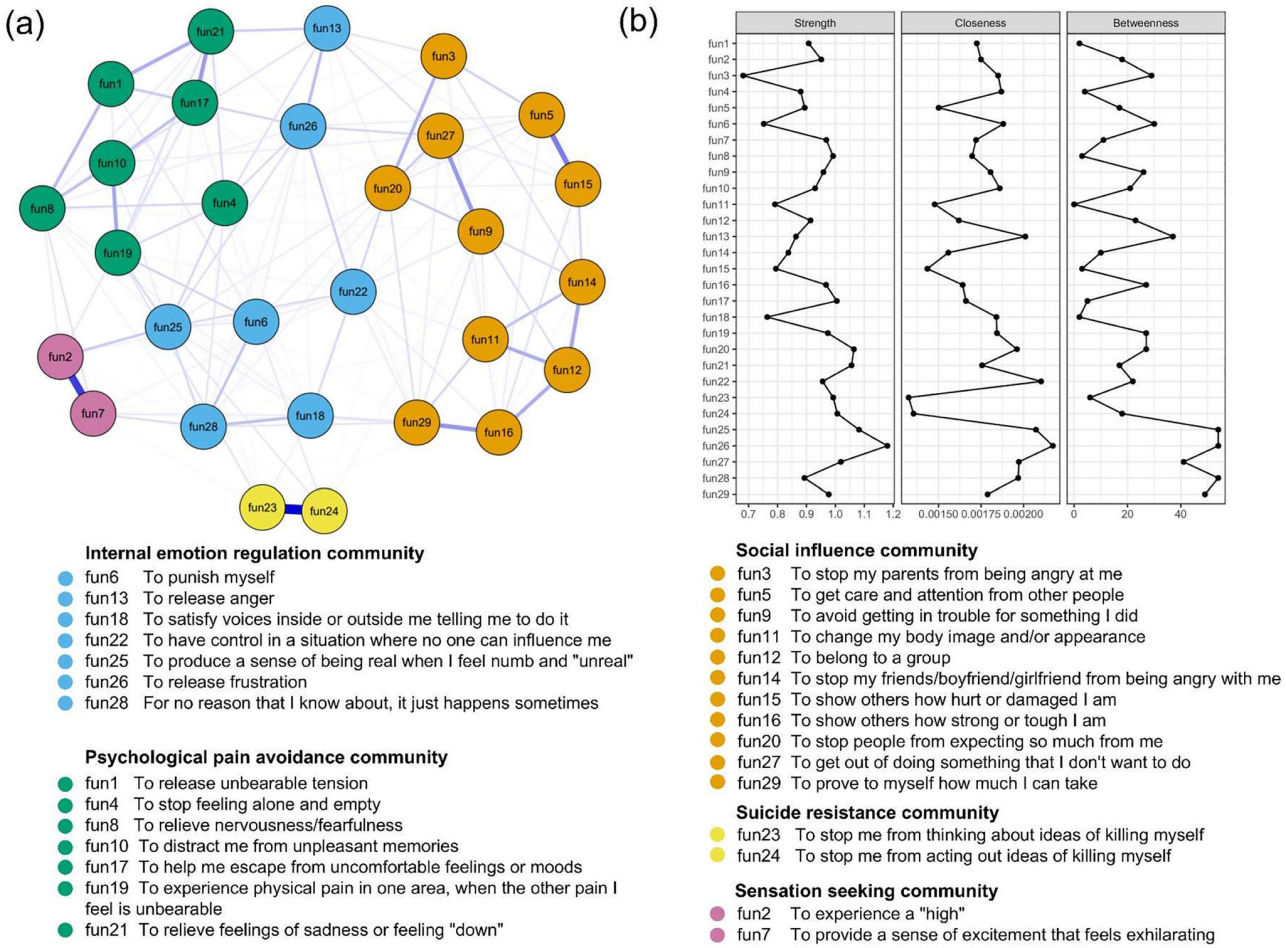
The NSSI functions network and the centrality analysis graphs of each node. This figure illustrates the network structure of non‐suicidal self‐injury (NSSI) functions and the centrality analysis of individual nodes within the network. The analysis provides insights into the interconnectedness of NSSI functions and highlights the relative importance of each function in the overall network. (a) The network included 29 nodes, and five stable communities were detected, whose names were internal emotion regulation (IER), psychological pain avoidance (PPA), social influence (SI), suicidal resistance (SR), and sensation seeking (SS). The thickness of the edge between nodes indicates their correlation. (b) The centrality of each node in the whole network was reflected by three indices. Strength represents the sum of the absolute values of edges’ weights of other nodes connected to the node. Closeness refers to the reciprocal of the sum of the shortest paths from the node to other nodes. Betweenness represents the number of times the node appears on the shortest path between two other nodes. The larger the values of the above indices, the stronger the node centrality and the more important its role in the network.

We included 29 functional items from OSI‐F as nodes in the whole‐sample network. Figure 1a showed the NSSI functions network and Supplementary Table S1 listed the representations of nodes. Five stable communities were detected, and we named these communities based on the functional items represented by the nodes and the NSSI functions proposed in previous research (Klonsky 2007).

Internal emotion regulation (IER), including 7 nodes in blue, was defined as regulating negative emotions such as frustration, anger, and numbness. Psychological pain avoidance (PPA), including 7 nodes in green, was defined as the avoidance or release of psychological pains such as unbearable pressure, unpleasant memories, and unbearable pain. Social influence (SI), including 11 nodes in orange, summarized the interpersonal influences such as gaining attention or avoiding things one doesn't want to do. Suicide resistance (SR), including 2 nodes in yellow, indicated the function of preventing suicidal ideation or committing suicide. Sensation seeking (SS), including 2 nodes in purple, meant the function of generating exhilaration or excitement.

The thickness of the edge between nodes indicated their correlation. Within the whole model, the strongest edges included the connection between “stop suicidal thought‐fun 23” and “stop suicidal attempt‐fun 24”, which belonged to the SR community. The second strongest connection was between “experience a high‐fun 2” and “provide excitement‐ fun 7”, which belonged to the SS community. Within the SI community, “get attention‐fun 5” and “show hurt‐fun 15” showed the strongest correlation.

The ranking of strength within the whole NSSI functions network model showed that “release frustration‐fun 26” had the highest strength value of 1.18, followed by “eliminate numbness‐fun 25”, suggesting that these functions were the most influential in the model (Figure 1b). Referring to the closeness, “release frustration‐fun 26” showed the top value of 0.002, followed by “have control ‐fun 22”. When looking at the betweenness, “release frustration‐fun 26”, “for no reason‐fun 28”, and “reduce numbness‐fun 25” got the highest value of 54. The above nodes all belonged to the IER community, indicating that IER, particularly “release frustration‐fun 26”, had strong centrality and played a critical role in the NSSI functions network (value of centrality including strength, closeness, and betweenness were shown in Supplementary Table S2).

#### 3.2.2 Network Accuracy and Stability

The CS‐coefficient of edge and strength generated by the case‐dropping bootstrap method was 0.517 and 0.438, respectively, indicating that the stability of both indices was acceptable. The graphical results for the accuracy of edge weights were shown in Supplementary Figure S1. Generally, the widths of the bootstrapped 95 % CIs for edge weights were large, and some of the lower absolute edge weights did not significantly differ among edges, indicating that the weights of these edges should be interpreted with caution. In addition, the stability of strength and closeness dropped steadily and indicates that the orders of nodes indicated by strength and closeness were relatively stable. The stability of betweenness is slightly worse than strength and closeness. The graphical results of the stability of the centrality metrics were shown in Supplementary Figure S2.

### 3.3 Related Psychosocial Factors of NSSI Functions

#### 3.3.1 Correlations Between NSSI Functions and Psychosocial Features

The heatmap showed the correlations between five NSSI functions and psychosocial features (Supplementary Figure S3). We found that almost every dimension of adolescents’ life events was significantly correlated with each NSSI function, especially interpersonal stress, whose correlation coefficients varied from 0.13 to 0.41. As for childhood trauma, emotional and physical mistreatments were positively correlated with NSSI functions except SS (0.16 ≤ *r* ≤ 0.30). The sexual abuse was associated with IER only (*r* = 0.20). The physical negligence had weak correlations with IER (*r* = 0.15) and SI (*r* = 0.13). In terms of family functions, communication, affective involvement, and general functioning were correlated with PPA mildly (0.12 ≤ *r* ≤ 0.15). Affective involvement also had a weak correlation with IER (*r* = 0.12). The personality trait of neuroticism had the strongest correlation with IER (*r* = 0.36), followed by PPA (*r* = 0.29), SI (*r* = 0.23), and SS (*r* = 0.14). While extraversion was negatively correlated with IER (*r* = −0.14) and SR (*r* = −0.13), similar correlations were shown between agreeableness and SI (*r* = −0.31) and SS (*r* = −0.14).

#### 3.3.2 Stepwise Regression of Five NSSI Functions

**FIGURE 2:**
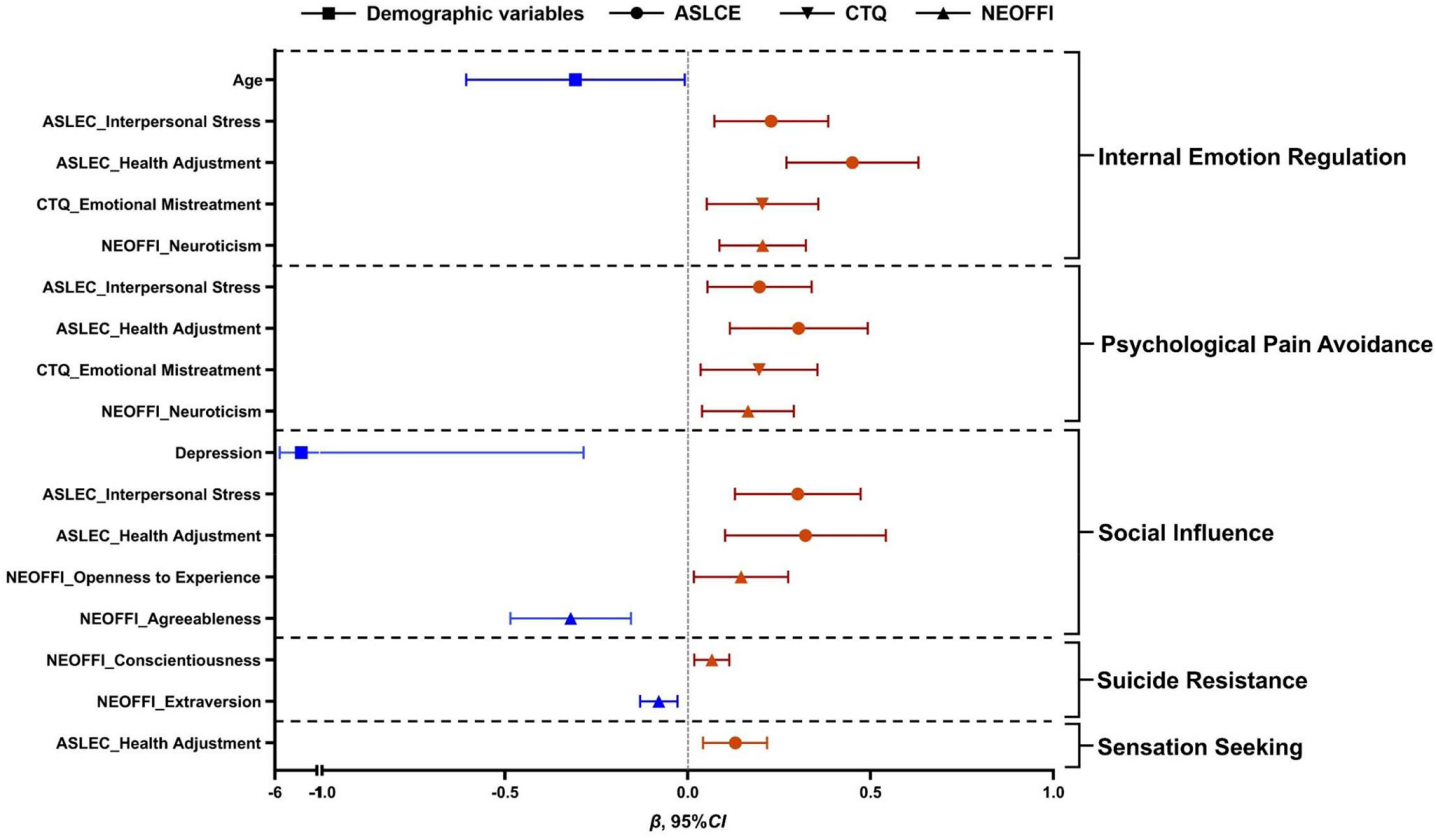
The forest plot of step‐wise linear regression of five NSSI functions. This figure presents the results of step‐wise linear regression analyses examining the associations between psychosocial factors and five non‐suicidal self‐injury (NSSI) functions. The covariates included age, gender, and dummy variables for diagnosis. The plot highlights the beta coefficients and their 95% confidence intervals (CIs) for each significant variable across the five NSSI functions. Positive coefficients were shown in red, and negative coefficients were shown in blue. ASLEC refers to the Adolescent Self‐Rating Life Events Checklist, NEOFFI refers to the NEO Five Factor Inventory, CTQ refers to the Childhood Trauma Questionnaire.

We conducted five stepwise regression analysis with each NSSI function as dependent variable, psychosocial factors significantly correlated with NSSI functions as independent variables, and gender, age, and dummy variables of diagnosis as covariates (Figure 2, Supplementary Table S3). The results revealed associations between IER function with age (*β =* −0.307; 95% confidence interval [*CI*], −0.606∼−0.008; *p * = 0.044), ASLEC‐health adjustment (*β =* 0.450; 95% CI, 0.270∼0.631; *p* < 0.001), interpersonal stress (*β =* 0.228; 95% CI, 0.073∼0.384; *p * = 0.004), neuroticism (*β =* 0.205; 95% CI, 0.087∼0.323; *p * =  0.001), and CTQ‐emotional mistreatment (*β =* 0.204; 95% CI, 0.052∼0.357; *p * = 0.009). The higher these scores, the more patients harm themselves for IER. In addition, the younger the age, the more likely the patients engaged in NSSI due to IER.

As for PPA function, the results showed that ASLEC‐health adjustment (*β =* 0.303; 95% CI, 0.115∼0.492; *p * = 0.002), interpersonal stress (*β =* 0.196; 95% CI, 0.054∼0.339; *p * = 0.007), neuroticism (*β =* 0.165; 95% CI, 0.039∼0.290; *p * =  0.010), and CTQ‐emotional mistreatment (*β =* 0.195; 95% CI, 0.035∼0.355; *p * = 0.017) could positively predicted it. The four factors were significantly associated with not only IER but also PPA.

Three factors were positively associated with the NSSI function of SI, and two factors were negatively associated with SI. The three factors were ASLEC‐health adjustment (*β =* 0.322; 95% CI, 0.102∼0.542; *p =* 0.004), interpersonal stress (*β =* 0.301; 95% CI, 0.129∼0.473; *p * = 0.001) and openness to experience (*β =* 0.146; 95% CI, 0.017∼0.275; *p * = 0.027). The two factors were depression diagnosis (*β =* −2.852; 95% CI, −5.420∼−0.285; *p * = 0.030) and agreeableness (*β =* −0.320; 95% CI, −0.485∼−0.155; *p* < 0.001).

The function of SR was associated with conscientiousness (*β =* 0.066; 95% CI, 0.018∼0.114; *p * = 0.007) and extraversion (*β =* −0.079; 95% CI, −0.130∼−0.028; *p * = 0.003). Only one factor could significantly predict the NSSI function of SS, ASLEC‐ health adjustment (*β =* 0.130; 95% CI, 0.042∼0.217; *p * =  0.004).

### 3.4 Mediation Analysis

**FIGURE 3:**
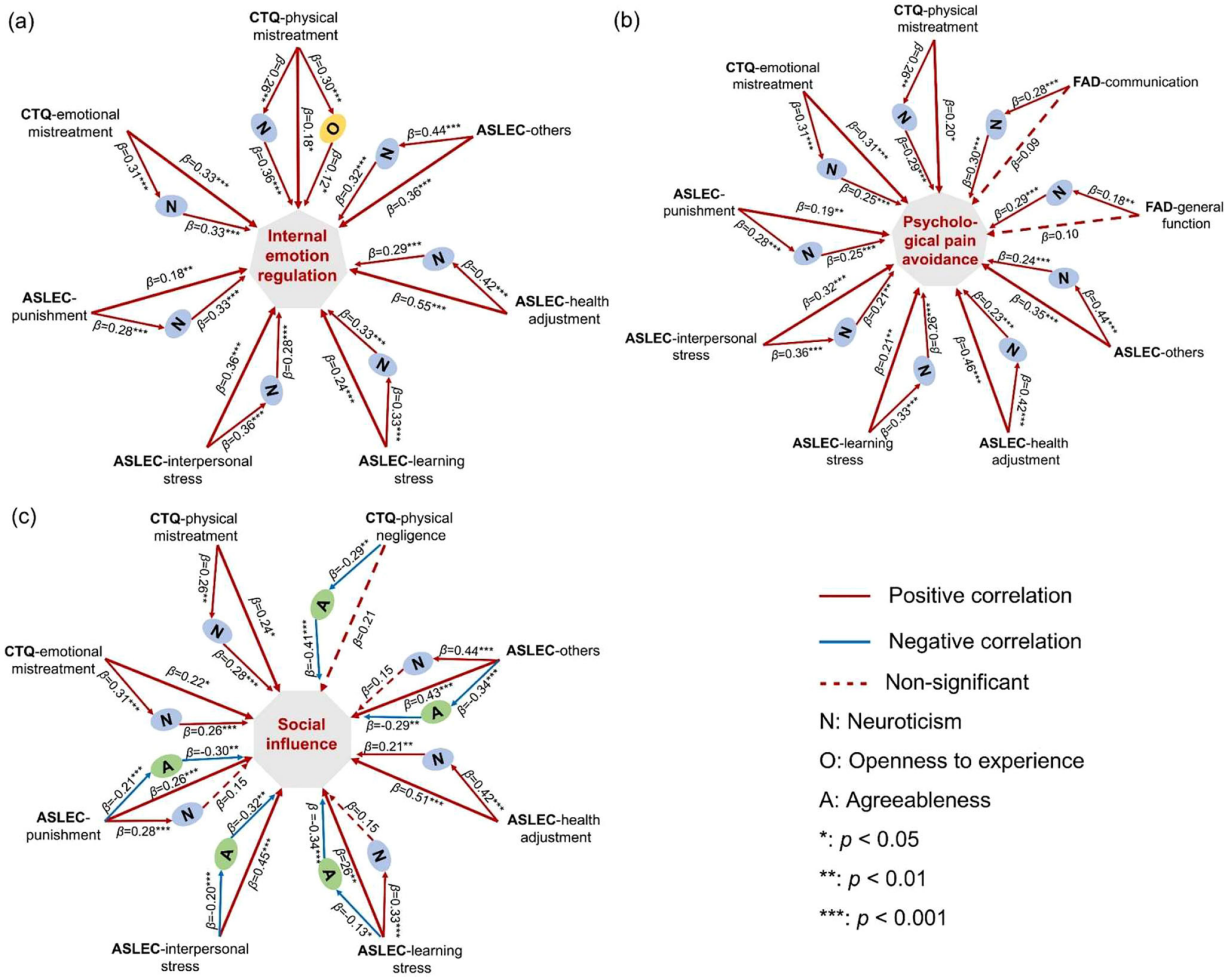
The mediation models between psychosocial factors and NSSI functions with personality traits as mediators. This figure illustrates the mediation effects of personality traits on the relationships between psychosocial factors and three distinct non‐suicidal self‐injury (NSSI) functions: internal emotion regulation (IER), psychological pain avoidance (PPA), and social influence (SI). Psychosocial factors were measured using the Adolescent Self‐Rating Life Events Checklist (ASLEC), Family Assessment Device (FAD), and Childhood Trauma Questionnaire (CTQ). (a) Seven mediation models showed seven psychosocial factors had both direct and indirect effects on the NSSI function of internal emotion regulation (IER), with significant mediation effects exerted by neuroticism (N). On the path from physical mistreatment to IER, openness to experience (O) played a parallel mediation effect with N. (b) Nine mediation models showed nine psychosocial factors had both direct and indirect effects on the NSSI function of psychological pain avoidance (PPA), with significant mediation effects exerted by neuroticism (N). There were two totally mediated effects exerted by N on the path from communication in FAD to PPA, as well as the path from general function in FAD to PPA. (c) Eight mediation models showed eight psychosocial factors had both direct and indirect effects on the NSSI function of social influence (SI), with significant mediation effects exerted by neuroticism (N) or agreeableness (A) or both the two. On the path from physical negligence in CTQ to SI, personality A played a total mediation effect. Notably, A had negative correlations with all psychosocial factors and SI.

To construct mediation models, we selected variables that showed significant pairwise correlations among psychosocial factors, personality traits, and NSSI functions. The significant results are detailed below (Figure 3). For the NSSI functions of sensation seeking (SS) and suicidal resistance (SR), no meaningful mediating effects were identified in the current analysis.

#### 3.4.1 Internal Emotion Regulation

Our analysis revealed that IER is influenced by both direct and indirect factors. Directly, IER was significantly affected by emotional mistreatment (*β* = 0.33, *p <* 0.001) and physical mistreatment (*β* = 0.18, *p =* 0.04) from the CTQ, as well as ASLEC, including punishment (*β* = 0.18, *p =* 0.003), interpersonal stress (*β* = 0.36, *p <* 0.001), learning stress (*β* = 0.24, *p <* 0.001), health adjustment (*β* = 0.55, *p <* 0.001), and others (*β* = 0.36, *p <* 0.001). Indirectly, neuroticism (N) served as a significant mediator across these pathways, while openness to experience (O) mediated the path from physical mistreatment to IER (Supplementary Table S4). These findings underscore the centrality of IER in the NSSI functional network, driven by adverse life events and modulated by specific personality traits.

#### 3.4.2 Psychological Pain Avoidance

PPA demonstrated the broadest range of influences among all NSSI functions. Directly, PPA was significantly influenced by emotional mistreatment (*β* = 0.31, *p <* 0.001) and physical mistreatment (*β* = 0.20, *p =* 0.028) from the CTQ, as well as ASLEC, including punishment (*β* = 0.19, *p =* 0.002), interpersonal stress (*β* = 0.32, *p <* 0.001), learning stress (*β* = 0.21, *p =* 0.006), health adjustment (*β* = 0.46, *p <* 0.001), and others (*β* = 0.35, *p <* 0.001). Indirectly, neuroticism (N) partially mediated the effects of CTQ and ASLEC on PPA and totally mediated the effects of FAD on PPA, reinforcing its importance in avoiding or releasing psychological pains (Supplementary Table S4).

#### 3.4.3 Social Influence (SI)

SI, a socially oriented NSSI function, showed distinct pathways. Directly, SI was significantly influenced by emotional mistreatment (*β* = 0.22, *p =* 0.022) and physical mistreatment (*β* = 0.24, *p =* 0.027) from the CTQ, as well as ASLEC, including punishment (*β* = 0.26, *p <* 0.001), interpersonal stress (*β* = 0.45, *p <* 0.001), learning stress (*β* = 0.26, *p =* 0.003), health adjustment (*β* = 0.51, *p <* 0.001), and others (*β* = 0.43, *p <* 0.001). Indirectly, neuroticism (N) mediated the effects of emotional mistreatment, physical mistreatment, and health adjustment on SI, while agreeableness (A) mediated the effects of interpersonal stress on SI. Agreeableness also totally mediated the effect of physical negligence on SI. Furthermore, on the path of influence from punishment, learning stress, and others to SI, neuroticism (N) and agreeableness (A) have played parallel mediating roles (Supplementary Table S4). These findings highlight the interpersonal nature of SI, with personality traits playing a significant role in its expression.

## 4 Discussion

This study provides critical insights into the functional network of NSSI, highlighting its multifaceted nature and complex interplay of psychological and environmental factors. IER was identified as a core function of NSSI, directly influenced by emotional and physical mistreatment as well as adolescent life stressors, with neuroticism serving as a key mediator and openness to experience mediating specific pathways. PPA emerged as the most broadly influenced function, strongly associated with both family functioning and stressors. The social influence functions, while less central, were uniquely linked to interpersonal dynamics, with agreeableness significantly mediating these effects. Neuroticism mediated the most number of pathways, underscoring its pivotal role in the underlying mechanisms of NSSI. These findings underscore the heterogeneity and overlapping nature of NSSI functions and deepen our understanding of how personality traits and psychosocial factors interact to shape different NSSI functions.

### 4.1 Internal Emotion Regulation: Central Role in NSSI Functions

This study highlights the pivotal role of IER within the functional network of NSSI. The “releasing frustration” in IER consistently demonstrated the highest centrality across strength, closeness, and betweenness measures, highlighting its dominance within the network. This finding supports prior research that identifies NSSI as a maladaptive coping mechanism for managing intense negative emotions, such as distress, frustration, and emotional numbness (Klonsky and Glenn 2009; Nock 2009). Individuals frequently report using NSSI to temporarily alleviate overwhelming internal states, suggesting that emotional dysregulation is not only a key trigger but also a sustaining factor in NSSI (K. L. Gratz 2001; Hooley and Franklin 2018). Neuroticism, a personality trait strongly linked to emotional instability, emerged as a significant mediator in pathways leading to IER, further reinforcing its centrality in the psychosocial mechanisms underlying NSSI behaviors (Cyders and Smith 2008; Yan and Yue 2023).

The implications of IER's centrality for clinical practice are profound and multifaceted. First, dialectical behavior therapy (DBT), which targets emotional dysregulation, effectively reduces NSSI by teaching adaptive coping strategies like mindfulness and distress tolerance (May et al. 2016). Tailoring DBT to improve emotional awareness and regulation could break the maladaptive feedback loop sustaining NSSI. Second, the strong connection between IER and childhood emotional mistreatment highlights the need for trauma‐informed approaches in NSSI treatment. These interventions address deep emotional wounds that drive self‐injury and help individuals develop healthier strategies for managing internal distress (van der Kolk 2014). Finally, the findings highlight the importance of early interventions to mitigate emotional dysregulation during adolescence, potentially reducing neuroticism (Jiang et al. 2024). By addressing the core mechanism of IER, clinicians can reduce the recurrence of self‐injury and promote long‐term emotional resilience in individuals with NSSI.

### 4.2 Psychological Pain Avoidance: Navigating Relational and Emotional Stressors

PPA involves escaping or releasing negative mental states such as unbearable pressure, unpleasant memories, and unbearable pain. This aligns with the experiential avoidance model, a model that was grounded in research on emotions and deliberate self‐harm, which posits NSSI as an escape from aversive emotional experiences (Chapman et al. 2006). Experiential avoidance refers to avoid any negative internal experiences or avoiding triggering negative experiences. NSSI is caused by ineffective emotional response, which can help individuals avoid aversive internal experiences such as thoughts, memories, and somatic feelings, although there are obvious negative consequences. Repeated relief through negative reinforcement strengthens the association between negative experience and NSSI, making NSSI an automatic coping response over time. Our findings highlight this function's broad range of influencing factors, including emotional and physical mistreatment, interpersonal stress, academic pressure, and family dysfunction, particularly poor communication and impaired overall functioning. Neuroticism emerged as a key mediator in these pathways, underscoring its role in amplifying external stress responses. Compared to previous research (Peel‐Wainwright et al. 2021), our study deepens the understanding of PPA by highlighting how the interplay between personality and relational stressors sustains this maladaptive coping mechanism. Clinically, PPA's reliance on external triggers and maladaptive coping mechanisms highlights the need for targeted interventions. DBT and Acceptance and Commitment Therapy (ACT) address emotional dysregulation (Hayes et al. 2012), and systemic family interventions focus on relational stressors(Weisz and Kazdin 2014), offering a comprehensive approach to reducing NSSI behaviors.

### 4.3 Social Influence: The Interpersonal Engine of NSSI

Social Influence (SI) captures the interpersonal motivations driving NSSI, such as avoiding conflict and expressing emotional distress. Our findings reveal its direct links to physical neglect, punishment, interpersonal stress, academic challenges, health adjustment, and other life events. Neuroticism and agreeableness emerged as pivotal mediators in these pathways, highlighting how external relational stressors and intrinsic personality traits intersect to sustain self‐injurious behaviors (Lüdtke et al. 2017). The strong connection between fun 5 (“to get care and attention from other people”) and fun 15 (“to show others how hurt or damaged I am”) further emphasizes the role of unmet relational needs in driving SI. Adolescents with low agreeableness often struggle with empathy and cooperation, heightening their risk of conflict‐laden relationships that exacerbate distress (J. M. Wang et al. 2017; You et al. 2016). This aligns with evidence highlighting the interpersonal context of mentalizing and relational patterns in perpetuating maladaptive behaviors (Bateman and Fonagy 2010). These findings further highlight the importance of interpersonal processes in maintaining the social influence function of NSSI. Interpersonal psychotherapy (IPT) can address relational deficits by enhancing communication, resolving conflicts, and strengthening social support networks (Weissman et al. 2000). Moreover, promoting high agreeableness through tailored approaches may reduce interpersonal challenges and foster healthier relationships (Bucher et al. 2019).

### 4.4 Suicide Resistance: A Paradoxical Protective Mechanism

SR highlights the paradoxical use of NSSI as a protective mechanism against suicidal ideation, where individuals engage in self‐injury to temporarily divert from or control overwhelming thoughts of self‐harm (Klonsky et al. 2013). In our study, SR was defined by the strongest connection between the functions “to stop me from thinking about ideas of killing myself” (fun 23) and “to stop me from acting out ideas of killing myself” (fun 24), reinforcing the notion that NSSI can serve as a maladaptive coping strategy to mitigate suicidal impulses. Personality traits, especially a high level of conscientiousness and a low level of extraversion emerged as critical influences on SR. High conscientiousness may provide a structured, albeit maladaptive, framework for managing distress; low extraversion suggests withdrawal from social support, increasing vulnerability to internalized stress (Carpenter and Trull 2013; K. L. Gratz et al. 2002). Clinically, Cognitive Behavioral Therapy for Suicide Prevention (CBT‐SP) effectively reduces suicidal ideation by fostering adaptive coping mechanisms and addressing the roots of self‐injury (Fox et al. 2015; Stanley et al. 2018). Promoting social engagement through group therapy or peer support networks may further offer resilience‐building alternatives to self‐injury (Cohen and Wills 1985; Van Orden et al. 2010). Integrating interpersonal and personality‐focused approaches enables clinicians to better address SR's role in NSSI and enhance long‐term outcomes.

### 4.5 Sensation Seeking: Arousal and Relief From Emotional Numbness

Sensation seeking (SS) reflects a function of NSSI, where individuals engage in self‐injury to achieve heightened arousal or escape emotional numbness. This behavior is strongly linked to impulsivity, novelty‐seeking, and external life stressors (Bender et al. 2011). Our findings underscore that SS is directly correlated with health adjustment, suggesting that difficulties in coping with health‐related stress may exacerbate the drive for sensation‐seeking behaviors, such as NSSI, by amplifying emotional and physical discomfort and highlighting the need for resilience‐focused interventions (Compas et al. 2017). SS positively correlates with neuroticism and negatively with agreeableness, aligning with evidence that impulsivity and poor emotional regulation drive risky behaviors, while higher agreeableness fosters adaptive social interactions and support (McHugh et al. 2019). These behaviors may align with an addiction‐like cycle, suggesting that NSSI as a sensation‐seeking mechanism could share similarities with addictive processes (Victor et al. 2012). Clinically, addressing SS requires strategies that satisfy the individual's need for stimulation while reducing reliance on self‐injury. Strategies such as creative interventions like adventure or music therapy and resilience‐focused approaches such as strength‐based counseling foster healthier coping mechanisms (Bowen et al. 2016; Calvete et al. 2022; Megranahan and Lynskey 2018).

### 4.6 The Chain of “Psychosocial Factors—Personality—Behavior”

This study reveals the intricate chain connecting psychosocial factors, personality traits, and behavioral expressions of NSSI. Childhood trauma, family dysfunction, and adolescent life stressors directly impact NSSI functions while shaping personality traits like neuroticism and agreeableness, which mediate these effects. For instance, emotional and physical mistreatments not only amplify IER and PPA functions but also increase neuroticism, a key trait linked to emotional instability and poor coping. Similarly, low agreeableness exacerbates SI by impairing empathy and interpersonal cooperation, reinforcing maladaptive behaviors to meet unmet relational needs. These findings underscore how environmental stressors and personality traits converge to create distinct psychosocial and behavioral pathways in NSSI, emphasizing the need for integrated interventions targeting external stressors and internal vulnerabilities. Clinically, this chain supports the need for tailored, multidimensional interventions. DBT addresses emotional dysregulation linked to IER and SS, while systemic family interventions target relational stressors underlying SI and PPA. IPT strengthens social connections to mitigate SI, and creative therapies like art or music therapy fulfill SS needs constructively. Resilience‐focused strategies, such as mindfulness and strength‐based counseling, provide additional support by enhancing emotional regulation and adaptive coping mechanisms. These approaches can be combined to interrupt maladaptive cycles, offering a pathway to sustainable behavioral and psychosocial change.

This study has several limitations that warrant consideration. First, its cross‐sectional design captures associations but the temporal sequence among psychosocial factors, personality, and NSSI functions could not be established. The mediation analyses should be interpreted as statistical mediation. Longitudinal or experimental studies are needed to verify these proposed pathways. Second, participants came from the inpatient department of a single center, which may limit the generalizability of our findings to community adolescents and outpatients. The sample had a female‐to‐male ratio of approximately 4:1, which may have limited our ability to detect potential sex differences, although we controlled for gender as a covariate in the statistical analyses whenever possible. The inclusion of participants with diverse psychiatric diagnoses also introduced diagnostic heterogeneity, which may have masked function‐specific differences across diagnostic groups. And the medication information of hospitalized patients was not controlled in the analysis. Third, the clinical recommendations for NSSI functions remain theoretical, as the study did not evaluate the efficacy of tailored interventions. Future research should quantify the core characteristics of each NSSI function and compare tailored versus non‐tailored treatment strategies. Fourth, cultural factors unique to East Asian populations may have influenced the findings, necessitating cross‐cultural studies to validate and generalize these results. Finally, reliance on self‐reported measures may introduce biases, such as underreporting or misinterpreting NSSI behaviors; future studies could mitigate this through multi‐informant and longitudinal designs. These limitations highlight the need for longitudinal, culturally sensitive, and function‐specific research to refine interventions and improve outcomes.

## 5 Conclusions

In conclusion, this study provides a comprehensive analysis of the functional network of NSSI, elucidating five distinct functions and their associated psychosocial factors. We proposed and tested the “psychosocial factors—personality—behavior” chain, which links stressors, personality, and NSSI behaviors. The findings offer a deeper understanding of the mechanisms underlying self‐injury. These insights pave the way for tailored, function‐oriented interventions, such as DBT for emotion regulation, systemic family therapy for relational stress, and resilience‐based strategies, enabling clinicians to systematically address the specific needs of individuals and improve treatment outcomes.

## Nomenclature

A: agreeableness
ACT: acceptance and commitment therapy
ASLEC: Adolescent Self‐Rating Life Events Checklist
CBT‐SP: cognitive behavioral therapy for suicide prevention
CI: confidence interval
CS‐coefficient: correlation stability coefficients
CTQ: Childhood Trauma Questionnaire
DBT: dialectical behavior therapy
DSM‐5: Diagnostic and Statistical Manual of Mental Disorders, Fifth Edition
FAD: Family Assessment Device
IER: internal emotion regulation
IPT: interpersonal psychotherapy
N: neuroticism
NEOFFI: NEO Five‐Factor Inventory
NSSI: non‐suicidal self‐injury
O: openness to experience
OSI‐F: Ottawa self‐injury inventory‐function subscale
PPA: psychological pain avoidance
SR: suicide resistance
SS: social influence
SS: sensation seeking

## Author Contributions

**Huiru Yan**: conceptualization, methodology, writing – original draft, data curation, formal analysis, writing – review and editing, visualization. **Hao Yan**: data curation, supervision, conceptualization, funding acquisition, project administration. **Mingzhu Li**: data curation. **Yuqi Ge**: data curation. **Yaoyao Sun**: methodology, writing – review. **Chuan Shi**: supervision. **Chao Wu**: writing – review and editing, methodology, funding acquisition. **Weihua Yue**: writing – review and editing, supervision, methodology, funding acquisition, project administration. **Xiao Zhang**: writing – review and editing, supervision, methodology, writing – original draft, funding acquisition, resources.

## Funding

This study was supported by the Beijing Research Ward Excellence Program (grant number BRWEP2024W074110113), National Natural Science Foundation of China (grant numbers 82441005 and 82330042), National Key R&D Program of China (grant number 2023YFE0119400), and Beijing Municipal Science & Technology Commission, Administrative Commission of Zhongguancun Science Park (grant number Z221100003522010).

## Ethics Statement

This study was approved by the ethics committee of the Peking University Sixth Hospital (2021‐39). All participants signed a written informed consent form.

## Conflicts of Interest

The authors declare no conflicts of interest.

## Supporting information




**Supplementary Materials**: brb371716‐sup‐0001‐SuppMat.docx

## Data Availability

The data that support the findings of this study are available from the corresponding author upon reasonable request.
