# Supplementary material for: Unraveling Non‐Suicidal Self‐Injury Functions Through Network Analysis and Revealing Their Triangular Relationship With Psychosocial Factors and Personality Traits
Source: Brain Behav. 2026 Aug 26;16(8):e71716. doi: 10.1002/brb3.71716 (PMC13510630; doi:10.1002/brb3.71716)
Supplement: Supplementary file 1 [file BRB3-16-e71716-s001.docx]

**Supplementary Material**

**Contents**

[**1. Supplementary texts for the assessment tools**	1](#_Toc235038881)

[**2. Supplementary figures**	3](#_Toc235038882)

[**3. Supplementary tables**	6](#_Toc235038883)

[**4. References**	13](#_Toc235038884)

**1. Supplementary texts for the assessment tools**

(1) ***Adolescent Self-Rating Life Events Checklist (ASLEC)*** (Wang, 1999) It consists of 27 items assessing whether individuals experienced specific life events during the past 12 months and the degree of impact of these events. The items are classified into six dimensions: punishment (7 items), loss (3 items), interpersonal stress (5 items), learning stress (5 items), health adjustment (4 items), and others (4 items). For each item, participants first indicate whether the event occurred (0 = no occurrence). If the event occurred, they rate the level of impact on a 5-point scale ranging from 1 (no impact) to 5 (extremely severe impact). Higher scores indicate exposure to more negative life events and greater perceived impact of these events.
(2) ***Family Assessment Device (FAD)*** (Wang, 1999) Family functioning was assessed using the FAD, which consists of 60 items evaluating seven dimensions: problem solving (6 items), communication (9 items), roles (11 items), affective responsiveness (6 items), affective involvement (7 items), behavior control (9 items), and general functioning (12 items). Each item is rated on a 4-point Likert scale ranging from 1 (strongly agree) to 4 (strongly disagree). The mean score of each dimension is calculated, with higher scores indicating poorer family functioning in that dimension. The FAD has demonstrated good reliability and validity among Chinese adolescents.
(3) ***Childhood Trauma Questionnaire (CTQ)*** (Zhao et al., 2005) Childhood trauma was assessed using the CTQ, which evaluates individuals’ experiences of adverse events before the age of 16 years. The CTQ consists of 28 items, including five dimensions: emotional abuse, physical abuse, sexual abuse, emotional neglect, and physical neglect. Each dimension contains 5 items, and each item is rated on a 5-point Likert scale ranging from 1 (never) to 5 (always). The scores of each dimension are calculated by summing the corresponding items, with higher scores indicating more severe childhood trauma experiences. In addition, three validity items are included to assess response validity and are not included in the total score calculation.
(4) ***NEO Five Factor Inventory (NEOFFI***)(Pervin et al., 2003) Personality traits were assessed using the NEOFFI, which evaluates five broad personality traits: neuroticism, extraversion, openness to experience, agreeableness, and conscientiousness. The NEO-FFI consists of 60 items, with 12 items assessing each personality trait. Each item is rated on a 5-point Likert scale ranging from 1 (strongly disagree) to 5 (strongly agree). Scores for each personality trait are calculated by summing the corresponding items, with higher scores indicating a higher level of the corresponding personality trait.

**2. Supplementary figures**

**Figure S1. Bootstrapped confidence intervals of estimated edge-weights.** The stability of edge weights across different bootstrap samples compared to the original sample were shown in this figure. The red line indicates the sample values and the gray area the bootstrapped confidence intervals. Each horizontal line represents one edge of the network, ordered from the edge with the highest edge-weight to the edge with the lowest edge-weight.

**Figure S2. Stability of the betweenness, closeness, and strength centrality index.** The red line depicts the betweenness changes after removing various proportions of data. The green line depicts the closeness changes after removing various proportions of data. The red line depicts the strength changes after removing various proportions of data.

**Figure S3. The heatmap of correlations between NSSI functions and environmental and psychological features.** This heatmap visualizes the correlation coefficients between non-suicidal self-injury (NSSI) functions and various environmental and psychological features, providing an intuitive overview of the strength and direction of these relationships. The environmental and psychological features were assessed using tools such as the Adolescent Self-Rating Life Events Checklist (ASLEC), Family Assessment Device (FAD), Childhood Trauma Questionnaire (CTQ), and NEO Five Factor Inventory (NEOFFI). Correlation significance levels are indicated by asterisks: *** (p < 0.001), ** (p < 0.01), and * (p < 0.05). Positive correlations are represented in shades of red, and negative correlations are displayed in shades of blue. Darker colors indicate stronger correlations, whether positive or negative.

**3. Supplementary tables**

| **Table S1. Nodes of NSSI Functions Network** | | |
| --- | --- | --- |
| **Community** | **Node name** | **Functions of NSSI (Ottawa self-injury inventory-function subscale)** |
| Internal emotion regulation | fun 6 | To punish myself |
|  | fun 13 | To release anger |
|  | fun 18 | To satisfy voices inside or outside me telling me to do it |
|  | fun 22 | To have control in a situation where no one can influence me |
|  | fun 25 | To produce a sense of being real when I feel numb and "unreal" |
|  | fun 26 | To release frustration |
|  | fun 28 | For no reason that I know about, it just happens sometimes |
| Psychological pain avoidance | fun 1 | To release unbearable tension |
|  | fun 4 | To stop feeling alone and empty |
|  | fun 8 | To relieve nervousness/fearfulness |
|  | fun 10 | To distract me from unpleasant memories |
|  | fun 17 | To help me escape from uncomfortable feelings or moods |
|  | fun 19 | To experience physical pain in one area, when the other pain I feel is unbearable |
|  | fun 21 | To relieve feelings of sadness or feeling "down" |
| Social influence | fun 3 | To stop my parents from being angry at me |
|  | fun 5 | To get care and attention from other people |
|  | fun 9 | To avoid getting in trouble for something I did |
|  | fun 11 | To change my body image and/or appearance |
|  | fun 12 | To belong to a group |
|  | fun 14 | To stop my friends/boyfriend/girlfriend from being angry with me |
|  | fun 15 | To show others how hurt or damaged I am |
|  | fun 16 | To show others how strong or tough I am |
|  | fun 20 | To stop people from expecting so much from me |
|  | fun 27 | To get out of doing something that I don't want to do |
|  | fun 29 | To prove to myself how much I can take |
| Suicide resistance | fun 23 | To stop me from thinking about ideas of killing myself |
|  | fun 24 | To stop me from acting out ideas of killing myself |
| Sensation seeking | fun 2 | To experience a "high" |
|  | fun 7 | To provide a sense of excitement that feels exhilarating |

| **Table S2 Value of centrality including strength, closeness, and betweenness** | | | |
| --- | --- | --- | --- |
| **Node** | **Strength** | **Closeness** | **Betweenness** |
| fun1 | 0.90777 | 0.00173 | 2 |
| fun2 | 0.95102 | 0.00175 | 18 |
| fun3 | 0.68099 | 0.00185 | 29 |
| fun4 | 0.88005 | 0.00187 | 4 |
| fun5 | 0.89386 | 0.00150 | 17 |
| fun6 | 0.75224 | 0.00188 | 30 |
| fun7 | 0.96841 | 0.00172 | 11 |
| fun8 | 0.99206 | 0.00170 | 3 |
| fun9 | 0.95855 | 0.00181 | 26 |
| fun10 | 0.93001 | 0.00186 | 21 |
| fun11 | 0.79116 | 0.00148 | 0 |
| fun12 | 0.91388 | 0.00162 | 23 |
| fun13 | 0.86368 | 0.00201 | 37 |
| fun14 | 0.83733 | 0.00156 | 10 |
| fun15 | 0.79436 | 0.00143 | 3 |
| fun16 | 0.96766 | 0.00164 | 27 |
| fun17 | 1.00472 | 0.00166 | 5 |
| fun18 | 0.76413 | 0.00184 | 2 |
| fun19 | 0.97403 | 0.00184 | 27 |
| fun20 | 1.06397 | 0.00196 | 27 |
| fun21 | 1.05577 | 0.00176 | 17 |
| fun22 | 0.95582 | 0.00210 | 22 |
| fun23 | 0.99240 | 0.00132 | 6 |
| fun24 | 1.00653 | 0.00135 | 18 |
| fun25 | 1.08176 | 0.00207 | 54 |
| fun26 | 1.17973 | 0.00217 | 54 |
| fun27 | 1.01890 | 0.00197 | 41 |
| fun28 | 0.89298 | 0.00197 | 54 |
| fun29 | 0.97702 | 0.00179 | 49 |

| **Table S3 The step-wise linear regression of five NSSI functions** | | | | | |
| --- | --- | --- | --- | --- | --- |
| **Dependent variables** | **Independent variables** | ***β*** | **95% *CI*** | ***p*-value** | **Adjusted *R^2^*** |
| Internal emotion regulation | |  |  |  | 0.305 |
|  | Age | -0.307 | -0.606~-0.008 | 0.044 |  |
|  | ASLEC_Health Adjustment | 0.450 | 0.270~0.631 | <0.001 |  |
|  | ASLEC_Interpersonal Stress | 0.228 | 0.073~0.384 | 0.004 |  |
|  | NEOFFI_Neuroticism | 0.205 | 0.087~0.323 | 0.001 |  |
|  | CTQ_Emotional Mistreatment | 0.204 | 0.052~0.357 | 0.009 |  |
| Psychological pain avoidance | |  |  |  | 0.194 |
|  | ASLEC_Health Adjustment | 0.303 | 0.115~0.492 | 0.002 |  |
|  | ASLEC_Interpersonal Stress | 0.196 | 0.054~0.339 | 0.007 |  |
|  | NEOFFI_Neuroticism | 0.165 | 0.039~0.290 | 0.010 |  |
|  | CTQ_Emotional Mistreatment | 0.195 | 0.035~0.355 | 0.017 |  |
| Social influence |  |  |  |  | 0.263 |
|  | Depression | -2.852 | -5.420~-0.285 | 0.030 |  |
|  | ASLEC_Health Adjustment | 0.322 | 0.102~0.542 | 0.004 |  |
|  | ASLEC_Interpersonal Stress | 0.301 | 0.129~0.473 | 0.001 |  |
|  | NEOFFI_Openness to Experience | 0.146 | 0.017~0.275 | 0.027 |  |
|  | NEOFFI_Agreeableness | -0.320 | -0.485~-0.155 | <0.001 |  |
| Sensation seeking | |  |  |  | 0.064 |
|  | ASLEC_Health Adjustment | 0.130 | 0.042~0.217 | 0.004 |  |
| Suicide resistance | |  |  |  | 0.081 |
|  | NEOFFI_Extraversion | -0.079 | -0.130~-0.028 | 0.003 |  |
|  | NEOFFI_Conscientiousness | 0.066 | 0.018~0.114 | 0.007 |  |

Note: NSSI, non-suicidal self-injury; ASLEC, Adolescent Self-Rating Life Events Check List; FAD, Family Assessment Device; CTQ, Childhood TraumaQuestionnaire; NEOFFI, NEO Five Factor Inventory.

| **Table S4 Significant mediation models of psychosocial factors (X) on NSSI functions (Y) mediated by personality (M)** | | | | | | | | | | | | | | | | | | |
| --- | --- | --- | --- | --- | --- | --- | --- | --- | --- | --- | --- | --- | --- | --- | --- | --- | --- | --- |
| **Mediation Models** | **X→Y** | | **M** | **X→M** | | **M→Y** | | **Total effect** | **Direct effect** | **Mediation effect** | | | | **Completely Standardized**  **Mediation effect** | | | | **Mediation proportion** |
|  | ***β*** | ***p*-value** |  | ***β*** | ***p*-value** | ***β*** | ***p*-value** | ***p*-value** | ***p*-value** | ***β*** | **Boot**  **SE** | **Boot**  **LLCI** | **Boot**  **ULCI** | ***β*** | **Boot**  **SE** | **Boot**  **LLCI** | **Boot**  **ULCI** |  |
| **Y: Internal emotion regulation** | | | | | | | | | | | | | | | | | | |
| X: CTQ-emotional mistreatment | 0.327 | <0.001 | N | 0.306 | <0.001 | 0.328 | <0.001 | <0.001 | <0.001 | 0.100 | 0.029 | 0.046 | 0.161 | 0.073 | 0.021 | 0.034 | 0.117 | 0.307 |
| X: CTQ-physical mistreatment | 0.185 | 0.044 | N | 0.260 | 0.003 | 0.362 | <0.001 | <0.001 | 0.044 | 0.094 | 0.037 | 0.028 | 0.170 | 0.061 | 0.023 | 0.018 | 0.106 | 0.299 |
|  |  |  | O | 0.305 | <0.001 | 0.120 | 0.047 |  |  | 0.037 | 0.021 | 0.002 | 0.087 | 0.024 | 0.014 | 0.001 | 0.056 | 0.116 |
| X: ASLEC- punishment | 0.184 | 0.003 | N | 0.282 | <0.001 | 0.333 | <0.001 | <0.001 | 0.003 | 0.094 | 0.023 | 0.053 | 0.143 | 0.095 | 0.024 | 0.054 | 0.146 | 0.508 |
| X: ASLEC-interpersonal  stress | 0.360 | <0.001 | N | 0.356 | <0.001 | 0.279 | <0.001 | <0.001 | <0.001 | 0.099 | 0.026 | 0.052 | 0.155 | 0.087 | 0.023 | 0.046 | 0.135 | 0.276 |
| X: ASLEC-learning  stress | 0.244 | <0.001 | N | 0.331 | <0.001 | 0.329 | <0.001 | <0.001 | <0.001 | 0.109 | 0.030 | 0.055 | 0.172 | 0.091 | 0.025 | 0.045 | 0.143 | 0.448 |
| X: ASLEC-health  adjustment | 0.555 | <0.001 | N | 0.418 | <0.001 | 0.292 | <0.001 | <0.001 | <0.001 | 0.122 | 0.032 | 0.064 | 0.191 | 0.076 | 0.020 | 0.040 | 0.117 | 0.220 |
| X: ASLEC-others | 0.360 | <0.001 | N | 0.436 | <0.001 | 0.320 | <0.001 | <0.001 | <0.001 | 0.140 | 0.035 | 0.075 | 0.213 | 0.088 | 0.022 | 0.047 | 0.133 | 0.388 |
| **Y: Psychological pain avoidance** | | | | | | | | | | | | | | | | | | |
| X: CTQ-emotional mistreatment | 0.308 | <0.001 | N | 0.306 | <0.001 | 0.253 | <0.001 | <0.001 | <0.001 | 0.078 | 0.028 | 0.029 | 0.139 | 0.060 | 0.020 | 0.021 | 0.100 | 0.252 |
| X: CTQ-physical mistreatment | 0.204 | 0.003 | N | 0.260 | 0.003 | 0.286 | <0.001 | 0.003 | 0.044 | 0.074 | 0.032 | 0.020 | 0.145 | 0.049 | 0.020 | 0.013 | 0.092 | 0.364 |
| X: ASLEC- punishment | 0.191 | 0.002 | N | 0.282 | <0.001 | 0.252 | <0.001 | <0.001 | 0.002 | 0.071 | 0.022 | 0.033 | 0.118 | 0.073 | 0.022 | 0.034 | 0.120 | 0.371 |
| X: ASLEC-interpersonal  stress | 0.320 | <0.001 | N | 0.356 | <0.001 | 0.212 | 0.001 | <0.001 | <0.001 | 0.076 | 0.026 | 0.029 | 0.132 | 0.066 | 0.023 | 0.026 | 0.115 | 0.236 |
| X: ASLEC-learning  stress | 0.211 | 0.006 | N | 0.331 | <0.001 | 0.258 | <0.001 | <0.001 | 0.006 | 0.086 | 0.028 | 0.036 | 0.146 | 0.072 | 0.023 | 0.031 | 0.121 | 0.405 |
| X: ASLEC-health  adjustment | 0.464 | <0.001 | N | 0.418 | <0.001 | 0.229 | <0.001 | <0.001 | <0.001 | 0.096 | 0.032 | 0.041 | 0.167 | 0.060 | 0.020 | 0.026 | 0.103 | 0.206 |
| X: ASLEC-others | 0.355 | <0.001 | N | 0.436 | <0.001 | 0.242 | <0.001 | <0.001 | <0.001 | 0.106 | 0.034 | 0.044 | 0.177 | 0.067 | 0.022 | 0.027 | 0.112 | 0.298 |
| X: FAD-communication | 0.093 | 0.290 | N | 0.279 | <0.001 | 0.297 | <0.001 | 0.049 | 0.290 | 0.083 | 0.033 | 0.030 | 0.157 | 0.058 | 0.022 | 0.021 | 0.107 | 0.895 |
| X: FAD-general  function | 0.096 | 0.112 | N | 0.183 | 0.001 | 0.291 | <0.001 | 0.015 | 0.112 | 0.053 | 0.020 | 0.019 | 0.097 | 0.054 | 0.019 | 0.020 | 0.095 | 0.555 |
| **Y: Social influence** | | | | | | | | | | | | | | | | | | |
| X: CTQ-emotional mistreatment | 0.223 | 0.022 | N | 0.306 | <0.001 | 0.264 | <0.001 | 0.002 | 0.022 | 0.081 | 0.029 | 0.029 | 0.142 | 0.050 | 0.018 | 0.018 | 0.087 | 0.363 |
| X: CTQ-physical mistreatment | 0.241 | 0.027 | N | 0.260 | 0.003 | 0.276 | <0.001 | 0.004 | 0.027 | 0.072 | 0.032 | 0.019 | 0.145 | 0.040 | 0.017 | 0.010 | 0.078 | 0.298 |
| X: CTQ-physical  negligence | 0.207 | 0.142 | A | -0.291 | 0.003 | -0.413 | <0.001 | 0.023 | 0.142 | 0.120 | 0.058 | 0.022 | 0.249 | 0.051 | 0.023 | 0.010 | 0.100 | 0.581 |
| X: ASLEC- punishment | 0.260 | <0.001 | N | 0.282 | <0.001 | 0.147 | 0.056 | <0.001 | 0.002 | 0.041 | 0.020 | 0.003 | 0.083 | 0.036 | 0.018 | 0.003 | 0.073 | 0.160 |
|  |  |  | A | -0.206 | <0.001 | -0.298 | 0.001 |  |  | 0.062 | 0.025 | 0.018 | 0.115 | 0.053 | 0.020 | 0.017 | 0.096 | 0.237 |
| X: ASLEC-interpersonal  stress | 0.453 | <0.001 | A | -0.204 | <0.001 | -0.323 | 0.001 | <0.001 | <0.001 | 0.066 | 0.026 | 0.020 | 0.123 | 0.049 | 0.018 | 0.016 | 0.088 | 0.146 |
| X: ASLEC-learning  stress | 0.258 | 0.003 | N | 0.331 | <0.001 | 0.151 | 0.053 | <0.001 | 0.003 | 0.050 | 0.026 | 0.002 | 0.101 | 0.036 | 0.018 | 0.001 | 0.073 | 0.194 |
|  |  |  | A | -0.133 | 0.025 | -0.344 | <0.001 |  |  | 0.046 | 0.026 | 0.004 | 0.104 | 0.033 | 0.018 | 0.003 | 0.072 | 0.177 |
| X: ASLEC-health  adjustment | 0.514 | <0.001 | N | 0.418 | <0.001 | 0.215 | 0.004 | <0.001 | <0.001 | 0.090 | 0.036 | 0.026 | 0.165 | 0.048 | 0.019 | 0.014 | 0.087 | 0.175 |
| X: ASLEC-others | 0.435 | <0.001 | N | 0.436 | <0.001 | 0.146 | 0.056 | <0.001 | <0.001 | 0.064 | 0.033 | 0.003 | 0.133 | 0.034 | 0.018 | 0.002 | 0.071 | 0.146 |
|  |  |  | A | -0.343 | <0.001 | -0.288 | 0.002 |  |  | 0.099 | 0.040 | 0.029 | 0.186 | 0.053 | 0.020 | 0.017 | 0.095 | 0.227 |
| Note: NSSI, non-suicidal self-injury; ASLEC, Adolescent Self-Rating Life Events Check List; FAD, Family Assessment Device; CTQ, Childhood Trauma Questionnaire; N, Neuroticism; O, Openness to Experience; A, Agreeableness. | | | | | | | | | | | | | | | | | | |

**4. References**

Pervin, L.A., John, O.P., Huang, X.T., 2003. Handbook of personality theory and research. East China Normal University Press, Shanghai.

Wang, X.D., 1999. Handbook of mental health assessment scales. Chinese Mental Health Journal.

Zhao, X.F., Zhang, Y.L., Li, L.F., Zhou, Y.F., 2005. Evaluation on reliability and validity of Chinese version of childhood trauma questionnaire. Chinese Journal of Clinical Rehabilitation. 209-211.
